# A Case Report of Obstructive Shock from an Esophageal Bolus Leading to Left Atrial Compression

**DOI:** 10.5811/cpcem.21220

**Published:** 2024-12-12

**Authors:** Sharmin Kalam, Sergio Marquez, Emmelyn J. Samones, Tammy Phan, Vi Am Dinh

**Affiliations:** Loma Linda University Medical Center, Department of Emergency Medicine, Loma Linda, California

**Keywords:** left atrial compression, esophageal mass, extracardiac compression, obstructive shock, case report

## Abstract

**Introduction:**

Obstructive shock results from reduced cardiac output due to physical blockage of blood flow, such as cardiac tamponade. Cardiac tamponade compresses cardiac chambers, particularly the left atrium, causing decreased end-diastolic volume and cardiac output. Rapid fluid accumulation within the pericardial sac is the usual cause. Transesophageal echocardiography provides clearer visualization of these structures than transthoracic ultrasound. This case underlines the impact of esophageal pathology on cardiac output and highlights ultrasound’s dynamic diagnostic utility alongside computed tomography.

**Case Report:**

A 64-year-old female with a history of colon cancer and peritoneal metastases status post colostomy presented with altered mental status and urinary symptoms. Laboratory evaluation was notable for leukopenia, hypoglycemia, elevated ammonia, and an abnormal urinalysis that was positive for urinary tract infection. She was initially admitted to the internal medicine service for sepsis secondary to urine as the source of infection. During her hospital stay, she developed hypotension, tachypnea, tachycardia, and complained of chest pressure. Point-of-care echocardiogram revealed compression of the left atrium by distended gastric and esophageal contents. A nasogastric tube was placed and suctioned partially digested food and liquid with improvement of her condition. Follow-up ultrasound showed improvement of compression and cardiac function.

**Conclusion:**

In evaluation of acute shock, multiple etiologies must be considered. In this case, the cause of reduced cardiac output was direct compression of the left atrium from an adjacent structure. Even with direct visualization and imaging, immediate history and patient-centered approach are still useful to complete the clinical picture and treat the reversible cause of undifferentiated shock.

## INTRODUCTION

Obstructive shock occurs when there is a physical blockage of blood flow that results in reduced cardiac output and hypoperfusion.^1^ Although the focus of this report is on pericardial tamponade, other etiologies of obstructive shock include massive pulmonary embolism, tension pneumothorax, vena cava compression, pulmonary compression, and aortic dissection.^1^ Cardiac tamponade is a life-threatening condition with a well-understood pathophysiology, necessitating prompt intervention. Clinical signs include hypotension, evidence of pulmonary edema, chest pain, dyspnea, tachypnea, muffled heart sounds, and jugular venous distention.

The hemodynamic compromise in cardiac tamponade is caused by compression of the cardiac chambers, particularly the left atrium and left ventricle, directly reducing end-diastolic volume and cardiac output.^2^ The etiology is often rapid accumulation of fluid within the pericardial sac, stemming from trauma, infection, malignancy, or other non-infectious causes.^1^–^3^ The close anatomical proximity of the distal esophagus to the left atrium is exploited by transesophageal echocardiography to provide direct visualization of the cardiac structures. This case re-emphasizes the direct physical relationship and potential impact of esophageal pathology reducing cardiac output. It also highlights the utility of ultrasound in providing dynamic diagnostic utility to supplement static computed tomography (CT).

## CASE REPORT

A 64-year-old female with a past medical history of colon cancer with peritoneal metastases and prior colostomy with both external and internal metastasis near her ostomy site was brought in by family for altered mental status. She had also been complaining of urinary symptoms, including frequency and urgency. On presentation, vital signs were temperature 98.2 °Fahrenheit, pulse 118 beats per minute, blood pressure 150/92 millimeters of mercury (mm Hg), respiratory rate 22 breaths per minute, and oxygen saturation 100% on room air. On physical examination, the patient was in mild distress and cachectic. She was unable to follow command. Her pupils were equal, round, and reactive to light with no apparent focal deficits. Her abdomen was soft and non-tender with an ostomy bag in place showing brown stool. Heart rate was tachycardic without murmurs and lungs were clear bilaterally.

Laboratory values revealed a white blood count of 4.74 x 10^9^ cells per liter (L) (reference range 4.8–11.8 x 10^9^ cells/L), a blood glucose of 53 milligrams per deciliter (mg/dL) (70–185 mg/dL) and an elevated ammonia level of 207 micromoles/L (μmol/L) (11–48 μmol /L). Additionally, her urinalysis showed moderate leukocytes, 13 white blood cells per high-powered field (HPF) (0–5 HPF), and bacteriuria. Two ampules of 50% dextrose were subsequently given with mild improvement of her mental status. Clinical suspicion of sepsis was made due to presence of altered mental status despite treatment of hypoglycemia. Infectious workup was obtained with the following results: wwhite blood cells 3.48 x 10^9^ per L, aspartate aminotransferase 36 units/L (U/L) (0–30 U/L), alanine aminotransferase 41 U/L (7–37 U/L), anion gap 14 (7–16), lactate 3.5 millimole per L (mmol/L) (0.5–20 mmol/L), no growth on blood cultures, and urine culture positive for *Enterococcus faecalis*. Intravenous ceftriaxone was ordered to treat urinary as source of infection.

**CPC-EM Capsule**

What do we already know about this clinical entity?

*Obstructive shock occurs when blood flow to the heart is obstructed, preventing it from pumping enough blood*.

What makes this presentation of disease reportable?

*Esophageal tamponade leading to obstructive shock is rare, with few reported cases of this association*.

What is the major learning point?

*Clinicians should have a broad differential diagnosis for acute presentations of shock, specifically for cardiac tamponade caused by a food bolus*.

How might this improve emergency medicine practice?

*Point-of-care ultrasound is a rapid and valuable tool to differentiate etiologies of shock, helping assess and guide appropriate treatment*.

**Image 1:**
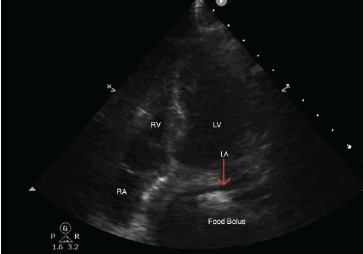
Apical 4-chamber cardiac view from point-of-care ultrasound showing a food bolus causing gastric outlet obstruction and external compression of the left atrium (arrow). *RA*, right atrium; *RV*, right ventricle; *LV*, left ventricle; *LA*, left atrium.

The following day, the patient required vasopressor support and was transferred to the intensive care unit (ICU). She was found to have a urine culture positive for Enterococcus as well as blood cultures positive for *Klebsiella pneumoniae*. Her ICU course was complicated by respiratory decompensation due to suspected fluid resuscitation and concurrent hypoalbuminemia resulting in large pleural effusions requiring a thoracentesis and high-flow nasal cannula (HFNC). She was weaned to two liters nasal cannula with aggressive diuresis and fluid restriction and subsequently downgraded. The following morning, shortly after breakfast, she developed tachypnea in the 40 breaths per minute range and was found to be hypotensive with systolic blood pressure in the 70 mm Hg range. She was placed on a non-rebreather mask at 15 L per minute (L/min), and 500 milliliters (mL) normal saline bolus was given with improvement of her systolic blood pressure to 105 mm Hg. She was started on HFNC at 30 L/min and 100% oxygen. In the ICU, she remained hypotensive with systolic blood pressures ranging from 90–100 mm Hg and tachycardia in the low 100s beats per minute but was otherwise alert and oriented with no concern for impending airway compromise. When asked what happened to her, she replied, “It was that shake I drank.” Point-of-care echocardiography was performed and revealed that the left atrium could not be clearly visualized secondary to anatomical compression by distended gastric as well as esophageal contents (Image 1).

**Image 2:**
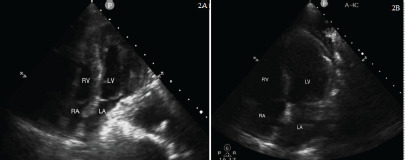
(2A) Apical 4-chamber cardiac view from point-of-care ultrasound of the left atrium immediately after nasogastric tube (NG) suction of approximately 600 milliliters of content. (2B) The same view of the left atrium after continued NG tube suctioning the following morning. *RA*, right atrium; *RV*, right ventricle; *LV*, left ventricle; *LA*, left atrium.

**Image 3:**
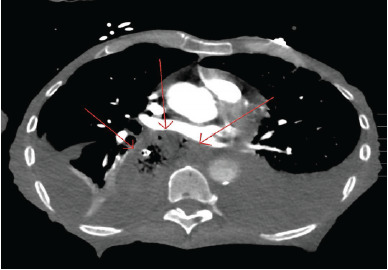
Transverse view from computed tomography angiography of the chest showing evidence of a distended esophagus with a large amount of intraluminal contents (arrows).

As a result of the ultrasound, a nasogastric (NG) tube was placed and immediately suctioned, yielding approximately 600 mL of partially digested food and liquid with subjective relief voiced by the patient, as well as clinical improvement (Image 2A). Her systolic blood pressure improved from 90 to low 100s mm Hg, and her HFNC was weaned to 14 L/min at 45% oxygen. A repeat ultrasound demonstrated visualization of the left atrium and improved cardiac function. Given the patient’s comorbidities, a CT angiogram of the chest was ordered to evaluate for possible pulmonary embolism (PE). This took place following point-of-care ultrasound (POCUS) and suctioning. No PE was found; however, a severely distended esophagus persisted with a large amount of intraluminal fluid (Image 3). The patient was made nil per os. The NG tube was also advanced to continuous suction overnight. A follow-up POCUS the next morning showed that the left atrium was no longer compressed (Image 2B). This correlated clinically with stable hemodynamic status as monitored with an arterial line.

## DISCUSSION

Obstructive shock is defined as physical obstruction of blood flow resulting in decreased cardiac output ultimately leading to tissue hypoperfusion. Prompt intervention is required for cardiac tamponade due to its life-threatening condition. It is believed that this patient experienced an accumulation of digestive material within the esophagus due to delayed gastric emptying and possibly a gastric outlet obstruction, leading to external compression of the left atrium. The relief of the left atrial obstruction with the NG tube suctioning seems to have reversed the patient’s shock. An esophagram performed three days later showed evidence of residual contrast within the distal esophagus and minimal entry into the stomach. The cause of the obstruction was believed to be related to her progressive metastatic disease. The patient was ultimately discharged home under hospice care.

This case highlights the importance of performing a rapid ultrasound for shock and hypotension exam in undifferentiated shock. The inferior vena cava was plethoric, which is consistent with an obstructive process. The rapid identification of left atrial compression allowed for immediate intervention with marked clinical improvement. Gastrointestinal pathology leading to obstructive shock is rare but has been shown in the setting of compartment syndrome or hepatic pathology from compression of central veins.^4^ Cases of similar presentations as the one mentioned above have been reported, and esophageal tamponade is an uncommon, yet known, phenomenon.^5^,^6^

A recent published report details a case of progressive achalasia leading to heart failure, successfully treated with esophageal dilation, underscoring the anatomic relationship between the mid esophagus and the left atrium.^7^ In the majority of the cases reviewed, there was a known history of progressive achalasia without any association with metastatic disease or outlet obstruction. One case report did describe acute heart failure with clinical evidence of hypoperfusion but with preserved hemodynamic stability.^8^ Additionally, while heart failure and pulmonary edema are mentioned, these cases did not describe acute shock in an ICU setting. Their lower acuity allowed for slower treatment options.^9^–^15^ This case represents an example of rapid diagnosis with POCUS allowing for a rapid intervention and reversal of a life-threatening condition.

## CONCLUSION

In evaluation of the patient with acute shock, multiple etiologies must be considered. In this case the cause of reduced cardiac output was direct compression of the left atrium by an adjacent distended esophagus. POCUS was important in diagnosing the condition, and the patient’s hemodynamic status improved with nasogastric suction of the esophageal contents.
